# Prioritizing mental and brain health in the global response to gender-based violence against women migrants and refugees

**DOI:** 10.3389/fpubh.2025.1567898

**Published:** 2025-03-07

**Authors:** Mohamed Taiebine

**Affiliations:** Euromed Research Center, Euro-Mediterranean University of Fez (UEMF), Fez, Morocco

**Keywords:** health, mental, migrant, women, violence, brain, gender-based, neuropsychological

Each year, December 10th marks the International Day for the Elimination of Violence Against Women and to raise awareness about this global hidden endemic and human rights issue. This day serves as a reminder of the urgent need to address all forms of violence against women, including physical, sexual, emotional, economic and mental abuse. It underscores the importance of collective action to create a world where women and girls can live free from fear, stigmatization and violence, and to promote gender equality while empowering them worldwide.

## Global engagement and guidelines

In the context of sustainable development goals (SDG) 3 to ensure good health and wellbeing (1), the Third Global Consultation on the Health of Refugees and Migrants, held in June 2023, resulted in the adoption of the Rabat Declaration (2) which signifies an increased commitment from the UN to improve the health of refugees and migrants through more inclusive national and international health policies. While the aforementioned declaration, endorsed by 30 member states, represents a positive step, it notably omits sufficient attention to the profound mental and brain health consequences for women migrants and refugees who are survivors of gender-based violence (GBV). Thus, this crucial gap undermines the declaration's potential impact, as GBV, encompassing a spectrum of abuses including sexual assault, domestic violence, and exploitation, inflicts severe and long-lasting trauma with significant neurological, neuropsychological and mental repercussions.

Similarly, the World Health Organization (WHO) global plan of action to strengthen the role of the health system within a national intersectoral response to address interpersonal violence, in particular against women, girls and children, provides a crucial framework for countries to develop and implement comprehensive, evidence-based strategies to prevent and respond to this pervasive issue (3). Within this roadmap, the care pathway outlined in the International Organization for Migration (IOM) (4) report serves as a crucial reference for a coordinated and effective response to these critical issues. This pathway is designed to be flexible and adaptable to various local contexts, ensuring that victims receive comprehensive support, starting from the initial reception and listening phase through to their social reintegration. It underscores the importance of collaboration among various stakeholders, including social, judicial, and medical services, to provide a tailored response that meets the unique needs of each victim. For instance, Iran has integrated registered refugees into its Universal Public Health Insurance program, granting them free access to a majority of primary healthcare services (5), while Morocco endorsed new laws and regulations to better integrate migrant in the healthcare system, providing them with free services, unlike Moroccan citizens (6).

## Mental and brain health issues of victims of GBV

Mental health and cerebral/brain health, though frequently regarded as synonymous, represent distinct yet interrelated concepts (7). Brain health pertains to the biological and physiological condition of the brain, including its structure, functionality, and developmental aspects. It can be shaped by various factors such as genetics, overall physical health, and lifestyle choices. Conversely, mental health encompasses a state of well-being that pertains to emotional, psychological, and social functioning, influencing our thoughts, feelings, and behaviors (7). The relationship between these constructs is reciprocal. For instance, in addressing GBV against women migrants and refugees, it is essential to consider both the potential repercussions on their brain health, including the neurological effects of trauma, and their mental health, which may manifest as conditions like post-traumatic stress disorder (PTSD), anxiety, and depression (8, 9).

The repercussions of GBV can adversely affect women's cognitive abilities, emotional stability, and interpersonal relationships. Additionally, the stress associated with displacement, coupled with insecurity and inadequate healthcare access, can worsen pre-existing mental health issues or trigger new ones (8). This is particularly concerning given the high prevalence of GBV among displaced populations and its devastating impact on mental wellbeing (9). For instance, a study of female urban refugees in Kampala found lifetime prevalence rates of 77.5% for experiencing any violence, with 92% showing symptoms of depression and 71.1% showing post-traumatic stress disorder symptoms (10).

Similarly, there is a concerning silent epidemic of online GBV which requires prioritizing mental and brain health across high, low, and middle-income countries and adds another layer of risk for migrant and refugee women's mental health. This later can be severe, with a dose-response relationship observed between exposure to violence and mental illness among refugees (11). Research indicates that refugee women face a higher prevalence of mental illness compared to general populations in host countries (12). This vulnerability is exacerbated by the multiple stressors associated with migration, including exposure to violence (11).

Furthermore, women and girls who have encountered GBV during their migration and displacement often endure considerable mental health challenges. These challenges encompass trauma-related disorders such as PTSD, depression, and anxiety (8, 9). The repercussions of these mental health issues can be profound and enduring, adversely affecting their overall wellbeing. This includes heightened susceptibility to further exploitation, difficulties in assimilating into new communities, and compromised parenting capabilities (13).

On the one hand, the mental and brain health impacts of GBV on refugee women are compounded by cultural factors and barriers to accessing care. Language barriers, stigma, and lack of culturally sensitive practices are major obstacles preventing refugee women from seeking mental health services in resettlement countries (14). On the other hand, acculturation may increase risk in some cases—one study found that Somali refugee women who reported greater English language ability also reported more experiences of intimate partner violence (15).

## Implications for policy and clinical practice

Addressing the mental and brain health needs of refugee women who have experienced GBV requires a holtistic as well as a salutogenic-based approach. Recommendations include providing culturally adapted care, involving mental healthcare through task-shifting, and increasing intervention intensity (15). Additionally, addressing gender stigma, improving health services, and strengthening policy responses are key strategies. Integrating mental health services into primary care, as recommended by the WHO, could improve access for migrant populations (16). However, cultural sensitivity is crucial, as perceptions and experiences of mental illness can vary significantly among refugee women (17). Developing assessment tools in local languages and providing formal training to healthcare providers could enhance mental health.

In terms of clinical neuropsychological screening, assessment and interventions, it is vital to ensure access to culturally sensitive mental health services, which should include both individual and group therapy, psychosocial support, and trauma-informed care. Additionally, addressing the underlying causes of GBV is imperative, which involves tackling discrimination, fostering gender equality, and empowering women and girls (18). Furthermore, strengthening social support systems is crucial, as this entails bolstering family and community networks and creating safe spaces for women and girls to heal and recover.

Despite these advancements, numerous barriers to healthcare access remain prevalent worldwide, encompassing structural, organizational, social, personal, and cultural challenges (18). Refugee women, in particular, often encounter discrimination, strained interactions with healthcare providers, and cultural misunderstandings within perinatal healthcare frameworks (19).

As emphasized by Hahn and Postmus (20), the implementation of culturally appropriate interventions, such as multilingual educational resources that explain various forms of violence and the process for seeking help, can significantly increase the likelihood of disclosure in clinical settings. Using mobile health and e-health approach (21) in anticipating pro-actively the neurocognitive and functional symptoms of GBV is crucial in balancing mental and brain health (22). Economic empowerment initiatives, including financial literacy programs and individual development accounts, have also shown promise in helping survivors attain economic independence (20).

Moreover, enhancing the collaboration among healthcare professionals, mental health experts, and legal authorities is essential to ensure that migrant women receive comprehensive and coordinated care. Additionally, the establishment of strong legal frameworks and policies is necessary to protect migrant women from GBV and facilitate their access to justice. Community-based support services, such as shelters and counseling centers, can provide vital assistance to survivors of GBV (20).

As nations prepare for the second High-Level Officials Meeting (HLOM) (23) which will take place on 16–17 December 2025, this event will build on stocktaking efforts at the country, regional, and global levels, to maintain momentum and assess progress against the implementation of the pledges made at the Global Refugee Forum (GRF) toward the advancement of the Global Compact on Refugees (GCR). Therefore, it is recommended that they prioritize mental and brain health in all initiatives and policies addressing GBV against women migrants and refugees, ensuring a holistic and comprehensive approach to their protection and integral wellbeing (7).

## Conclusion

There is an urgent need to implement neuropsychological and mental health services for migrant women who experienced GBV and are at risk of developing mental health disorders, such as anxiety, depression, and PTSD. From a life-span approach, such pervasive mental health conditions may trigger a myriad of neuropsychological and neurological vulnerabilities. Additionally, migrant women frequently encounter barriers when seeking help, as cultural stigma, language barriers, and fears of deportation can obstruct their access to vital services and support. By reducing the social and health determinants of GBV, including healthcare expenditures, productivity, and the strain on social welfare systems, women migrants and refugees will benefit from a smooth transition in their migration and life journey. A multidisciplinary and salutogenic strategy not only address immediate crises but also aim to prevent future incidents and facilitate proactive and sustainable rebuilding of individuals' mental, brain and social lives.
